# Physical function, ADL, and depressive symptoms in Chinese elderly: Evidence from the CHARLS

**DOI:** 10.3389/fpubh.2023.1017689

**Published:** 2023-02-22

**Authors:** Yumeng Yan, Yiqian Du, Xue Li, Weiwei Ping, Yunqi Chang

**Affiliations:** ^1^School of Public Health, Shanxi Medical University, Taiyuan, China; ^2^Department of Public Health and Preventive Medicine, Changzhi Medical College, Changzhi, China

**Keywords:** ADL disability, physical dysfunction, depressive symptoms, urban and rural elderly, CHARLS

## Abstract

**Background:**

Depressive symptoms are a serious public health problem that affects the mental health of older adults. However, current knowledge of the association between ADL disability and physical dysfunction and depressive symptoms in Chinese adults is insufficient. We intend to analyze the association between physical function, ADL, and depressive symptoms in older Chinese adults.

**Methods:**

The data obtained from the China Health and Retirement Longitudinal Survey (2015 and 2018) (CHARLS). This includes 3,431 in 2015 and 3,258 in 2018 over the age of 60. Comparing 2015 and 2018 data, multivariate logistic regression models were used to explore the relationship between physical function, ADL, and depressive symptoms in urban and rural older adults, adjusting for sociodemographic factors associated with depression in older adults.

**Results:**

The prevalence of depressive symptoms among older adults in China was 33.8 percent in 2015 and 50.6 percent in 2018. In baseline data from 2015 and 2018, residence, gender, marital status, drinking, physical function, ADL, and self-rated health were all found to be significantly associated with depressive symptoms in older adults. The differences in physical function, ADL and depressive symptoms among older adults in 2015 and 2018 were further analyzed based on urban and rural stratification. Both physical dysfunction and ADL disability were significantly associated with depressive symptoms in rural older adults in 2015 and 2018. And in urban areas, ADL was found to be significantly associated with depressive symptoms in urban older adults. Multivariate logistic regression analysis demonstrated that ADL disability was significantly associated with depressive symptoms among older adults in both urban and rural areas. Physical dysfunction was only significant in rural areas with depressive symptoms. The alpha level was instead set to 0.05 for all statistical tests.

**Conclusion:**

Rural, female, 60–70 years of age, primary school or below, married, non-smoking, non-drinking, physical dysfunction, ADL disability and self-rated poor health make-up a higher proportion of depressed older adults. ADL disability and physical dysfunction were more likely to be associated with depressive symptoms in rural Chinese older adults. Therefore, the physical and mental health of rural elderly should be of concern. The rural older adults should receive additional support from the government and society.

## 1. Introduction

As the number of older adults continues to increase, increasing attention was paid to the health problems of older adults (1). Depressive symptoms are common in older adults (1). There is evidence that depressive symptoms could be difficult to treat later in life (2). Besides, it might lead to reduced physical activities, lower the quality of life, and generate self-grief and even suicide (3). Studies showed that prevalence of depressive symptoms in Asian elderly was 7.8–46% (4). The overall prevalence of depressive symptoms was higher in Brazilian older adults (30.2%) than Chilean older adults (26.3%) (5). Furthermore, studies on older adults in China revealed that the prevalence differed between 13 and 41% (6). The disease burden of depressive symptoms in China had been on the rise and would continue to increase in the coming decades (7).

Researchers identified some as risk factors of depressive symptoms including female gender, somatic illness, cognitive impairment, functional disability, and history of depressive symptoms (8). Studies showed that reduced physical function in older adults was the main risk factor for developing depressive symptoms (9). A longitudinal community-based study reported that physical function independently predicts depressive morbidity in late-life (10). A study showed physical symptoms and poorer physical function reported increased depressive symptoms (11). At the same time, a decline in physical function leads to a loss of independence and consequent depressive symptoms. These studies demonstrated that those with the lowest levels of physical function carry the largest risk of onset of both depressive symptoms and anxiety over time (12). There was evidence that ADL disability may be a risk factor for depressive symptoms in previous studies (13, 14). ADL disability was associated with depressive symptoms and expanded psychological burden in older adults (15). An article on the level of depressive symptoms among elderly Turkish people, the findings indicated that ADL anticipated depressive symptoms among older adults (16). A study in South Korea reported that restriction of ADL, which means restriction of physical function, was also associated with early depressive symptoms. Lack of physical function leads to diminished social relations and depressive symptoms (17). In addition, economic, political, cultural, and other factors affect depressive symptoms differently. Depressive symptoms a financial burden on older adults and families. Studies demonstrated the medical expenses on depressive symptoms were 1.86 times that of non-depressed patients (18).

Currently, there was limited knowledge about the relationship between ADL disability and physical dysfunction and depressive symptoms in the Chinese older adults. In a prospective study of 2,713 Chinese older adults who completed interviews with the Chicago Chinese Aged Population Study, a significant relationship was discovered between depressive symptoms and the occurrence of functional disability (19). ADL disability was found to be a high-risk group for depressive symptoms in older adults in a study on changes in depressive symptoms levels in older Chinese (20). In a community-based study in Beijing, it was indicated that older adults with disabilities were more likely to experience depressive symptoms (21). Similarly, community-based research has linked ADL disability with increased risk of depressive symptoms in middle-aged and older Chinese adults (22). Data from one study showed that physical dysfunction in older silicosis patients was significantly associated with the prevalence of depressive symptoms (23). In addition, an analysis of factors influencing mental health in older Chinese adults showed that physical function and ADL were strongly associated with depressive symptoms in older adults (24).

In previous studies, depressive symptoms in older adults have mainly been studied in terms of ADL disability in a particular region or community. Our study was based on the China Health and Retirement Longitudinal Study (CHARLS), which was collected from respondents across the country. The sample of over-60 s used for the study was broader and more representative. Correspondingly, based on the above data, we mainly explore the relationship between physical function, ADL, and depressive symptoms. The purposes were the following: (1) To compare depressive symptoms prevalence in 2015 and 2018; (2) To study the influencing factors of depressive symptoms in older adults; (3) To evaluate the association between physical function, ADL, and depressive symptoms among urban and rural older Chinese adults.

## 2. Research methods

### 2.1. Data

The China Health and Retirement Longitudinal Study (CHARLS) is a large-scale interdisciplinary survey project hosted by the National Development Institute of Peking University and carried out by the China Social Science Survey Center of Peking University. It is high-quality microdata representing the households and individuals of middle-aged and older Chinese adults over the age of 45. CHARLS conducted surveys and interviews in 150 counties and 450 communities (villages) of 28 provinces (autonomous regions and municipalities) in 2011, 2013, 2015, and 2018, respectively. The CHARLS National Baseline Survey was launched in 2011 and followed for two years, with 23,000 respondents in 12,400 households. Data from 2015 and 2018 are used in this study. Seniors aged 60 and over were selected for the study. A total of 3,431 subjects were screened in 2015 and 3258 in 2018.

Ethical approval for data collection in CHARLS is obtained from the Biomedical Ethics Review Committee of Peking University. Peking University Public Data Management Agency agreed to our use of the data.

### 2.2. Depression

The Center for Epidemiological Studies Depression Scale (CES-D-10) was used to measure depressive symptoms in the CHARLS questionnaire. CES-D-10 was highly reliable and effective in successfully measuring depressive symptoms in middle-aged and older adults (25). Previous studies demonstrated that a score of 10 on the CES-D had reasonable levels of sensitivity (0.85) and specificity (0.80) in Chinese adults (26). The simplified scale consists of 10 questions with options as “rarely or none of the time (< 1 day), some or few times (1–2 days), occasionally or a moderate number of times (3–4 days), most of the time (5–7 days), the assignment value range was 0–3 points, total score was calculated. A higher score indicates greater symptoms of depression. A score of 10 and below was “no depressive symptoms” and assigned a value of 0; the score above 10 was “depressive symptoms” and assigned a value of 1 (24).

### 2.3. State of health

In CHARLS, self-rated health (SRH) was obtained by asking participants, “How do you feel about your health status?” SRH was transformed into two categories of variables, respectively, self-rated good health and self-rated poor health.

### 2.4. Physical function

The CHARLS questionnaire sets some physical function related questions, including: running or jogging 1 km, wandering 1 km, walking 100 meters, sitting in a chair for a long time and then standing up, ascending several floors continuously, bending over, bending knees or squat, stretch arms up along your shoulders, walk 100 meters to run or jog 1 km, pick up a tiny coin from the table, each answer for questions was divided into four responses as follows: (1) No difficulty; (2) Difficulty but still can be completed; (3) Difficulty and need help; (4) Unable to complete. If a subject reported difficulty with any of the 9 items, they were defined as having a physical dysfunction (24).

### 2.5. ADL

In CHARLS, the ADL scale was used to determine the disability of older adults. The ADL scale had good reliability and validity and was generally used in China and abroad (27). The ADL scale consists of 12 items: dressing, bathing, eating, getting into or out of bed, using the bathroom, controlling urination and defecation, doing household chores, cooking, shopping, making phone calls, taking medication, managing money. Each answer for questions was divided into 4 reactions as follows: (1) No, I do not have any difficulty; (2) I have difficulty but still can do it; (3) Yes, I have difficulty and need help; (4) I cannot do it (15). If a subject report having difficulty with any of the 12 items, then they were defined as having an ADL disability (24).

### 2.6. General demographic information

Covariates included gender, age, education level, marital status, address, smoking, drinking, physical exercise, and social activity. Gender included both males and females. Age was divided into 60–70, 71–80, 80 and above. Education levels were divided into primary school or below, middle school, high school or secondary school, and college or above. Marital status was classified as married or unmarried. Smoking, drinking, physical exercise, and social activity were divided into two groups: yes and no.

### 2.7. Statistical method

Excel 2019 was used to store and filter the data. IBM SPSS (version 22.0) was used for statistical analysis. Descriptive statistics were assigned to describe the demographic information of the participants. Continuous variables were presented as means and standard deviations. The categorical variables were presented as frequencies and percentages. The chi-squared test was used to compare categorical variables. Logistic regression was used when multiple variables were considered simultaneously. Multivariate logistic regression models were performed to compute the relationship between physical function, ADL, and depressive symptoms based on urban and rural stratification. Multivariate logistic regression analysis adjusted for sociodemographic confounding factors associated with depression in older adults. The statistical significance level was set at 0.05. Results were presented as odds ratios (ORs) and 95% confidence intervals (CIs).

## 3. Results

### 3.1. Study population

In 2015 and 2018, 3,431 and 3,258 older adults were included, respectively. The mean age of older adults was 66 [Standard Deviation (SD) = 7.041], and 63.1% of the participants were female, 27.7% resident in urban areas in 2015. The mean age of older adults was 68 [Standard Deviation (SD) = 6.563] years, and 68.4% of the participants were female, 31.2% resident in urban areas in 2018. The baseline characteristics were presented in Table 1.

**Table 1:** Demographic characteristics and depressive symptoms in elderly [*N* (%)].

**Variables**		**2015 (***n** =* 3,431)**				**2018 (***n** =* 3,258)**		
	**Total**	**No depressive symptoms (***n** =* 2,271)**	**Depressive symptoms (***n** =* 1,160)**	***P***	**Total**	**No depressive symptom (***n** =* 1,608)**	**Depressive symptoms (***n** =* 1,650)**	***P***
**Residence**								
Urban	945 (27.7)	685 (30.2)	260 (22.4)	< 0.001	1,016 (31.2)	567 (35.3)	449 (27.2)	< 0.001
Rural	2,486 (72.5)	1,586 (69.8)	900 (77.6)		2,242 (68.8)	1,041 (64.7)	1,201 (72.8)	
**Gender**								
Female	2,166 (63.1)	1,308 (57.6)	858 (74.0)	< 0.001	2,230 (68.4)	929 (59.6)	1,271 (77.0)	< 0.001
Male	1,265 (36.9)	963 (42.4)	302 (26.0)		1,028 (31.6)	649 (40.4)	379 (23.0)	
**Age (year)**								
60–70	2,249 (65.5)	1,450 (63.8)	799 (68.9)	0.001	1,817 (55.8)	884 (55.0)	933 (56.5)	0.440
71–80	923 (26.9)	624 (27.5)	299 (25.8)		1,162 (35.7)	577 (35.9)	585 (35.5)	
>80	259 (7.5)	197 (8.7)	62 (5.3)		279 (8.6)	147 (9.1)	132 (8.0)	
**Education level**								
Primary school or below	3,077 (89.7)	2,034 (89.6)	1,043 (89.9)	0.315	2,541 (78.0)	1,180 (73.4)	1,361 (82.5)	< 0.001
Middle school	253 (7.4)	163 (7.2)	90 (7.8)		420 (12.9)	250 (15.5)	170 (10.3)	
High school	79 (2.3)	56 (2.5)	23 (2.0)		231 (7.1)	134 (8.3)	97 (5.9)	
College or above	22 (0.6)	18 (0.8)	4 (0.3)		66 (2.0)	44 (2.7)	22 (1.3)	
**Marital status**								
Unmarried	795 (23.2)	491 (21.6)	304 (26.2)	< 0.001	776 (23.8)	332 (20.6)	444 (26.9)	< 0.001
Married	2,636 (76.8)	1,780 (78.4)	856 (73.8)		2,482 (76.2)	1,276 (79.4)	1,206 (73.1)	
**Smoking**								
No	2,485 (72.4)	1,554 (68.4)	931 (80.3)	< 0.001	2,949 (90.5)	1,450 (90.2)	1,499 (90.8)	0.511
Yes	946 (27.6)	717 (31.6)	229 (19.7)		309 (9.5)	158 (9.8)	151 (9.2)	
**Drinking**								
No	2,468 (71.9)	1,578 (69.5)	890 (76.7)	< 0.001	2,522 (77.4)	1,203 (74.8)	1,319 (79.9)	< 0.001
Yes	963 (28.1)	693 (30.5)	270 (23.3)		736 (22.6)	405 (25.2)	331 (20.1)	
**Physical exercise**								
No	360 (10.5)	226 (10.0)	134 (11.6)	0.148	272 (8.3)	112 (7.0)	160 (9.7)	0.005
Yes	3,071 (89.5)	2,045 (90.0)	1,026 (88.4)		2,986 (91.7)	1,496 (93.0)	1,490 (90.3)	
**Social activity**								
No	1,841 (53.7)	1,204 (53.0)	637 (54.9)	0.292	1,646 (50.5)	815 (50.7)	831 (50.4)	0.855
Yes	1,590 (46.3)	1,067 (47.0)	523 (45.1)		1,612 (49.5)	793 (49.3)	819 (49.6)	
**Physical function**								
Normal	526 (15.0)	381 (16.8)	135 (11.6)	< 0.001	1,016 (31.2)	646 (40.2)	370 (22.4)	< 0.001
Dysfunction	2,915 (85.0)	1,890 (83.2)	1,025 (88.4)		2,242 (68.8)	962 (59.8)	1,280 (77.6)	
**ADL**								
Normal	1,563 (45.6)	1,157 (50.9)	406 (35.0)	< 0.001	1,607 (49.3)	891 (55.4)	716 (43.4)	< 0.001
Disability	1,868 (54.4)	1,114 (49.1)	754 (65.0)		16,51 (50.7)	717 (44.6)	934 (56.6)	
**Self-rated health**								
Poor	1,956 (57.0)	1,172 (51.6)	784 (67.6)	< 0.001	2,677 (82.2)	1,220 (75.9)	1,457 (88.3)	< 0.001
Good	1,726 (45.4)	1,099 (48.4)	376 (32.4)		581 (17.8)	388 (24.1)	193 (11.7)	

### 3.2. Depressive symptoms in older adults

In 2015, 33.8% of older adults had depressive symptoms, which increased to 50.6% in 2018. In 2015, the proportion of older people with depressive symptoms in urban and rural areas was 27.5 and 36.2%, respectively, and will increase to 44.2 and 53.6% in 2018. Those who were unmarried, residence in rural, younger, lower education level, physical dysfunction, ADL disability, self-rated poor health was more likely to suffer from depressive symptoms in 2015 and in 2018 (Table 1).

### 3.3. Depressive symptoms in urban and rural

The prevalence of depressive symptoms in older adults was assessed in 2015 and 2018 respectively, and stratified by urban and rural areas at baseline. Based on 2015 data, physical dysfunction and ADL disability were all substantially related to depressive symptoms in rural older adults (Table 2). Based on data in 2018, physical dysfunction and ADL disability were all significantly related to depressive symptoms in urban and rural older adults (Table 3).

**Table 2:** Depressive symptoms based on urban and rural stratification of physical function and ADL in 2015 [N (%)].

**Variables**	**Urban (***n** =* 945)**			**Rural (***n** =* 2,486)**		
	**No depressive symptoms**	**Depressive symptoms**	***P***	**No depressive symptoms**	**Depressive symptoms**	***P***
**Total**	685 (72.5)	260 (27.5)		1,586 (63.8)	900 (36.2)	
**Physical function**			0.978			< 0.001
Normal	127 (18.5)	48 (18.5)		254 (16.0)	87 (9.7)	
Dysfunction	558 (81.5)	212 (81.5)		1,332 (84.0)	813 (90.3)	
**ADL**			0.003			< 0.001
Normal	405 (59.1)	126 (48.2)		752 (47.4)	280 (31.1)	
Disability	280 (40.9)	134 (51.5)		834 (52.6)	620 (68.9)	

**Table 3:** Depressive symptoms based on urban and rural stratification of physical function and ADL in 2018 [N (%)].

**Variables**	**Urban (***n** =* 1,016)**			**Rural (***n** =* 2,242)**		
	**No depressive symptoms**	**Depressive symptoms**	***P***	**No depressive symptoms**	**Depressive symptoms**	***P***
**Total**	567 (55.8)	449 (44.2)		1,041 (46.4)	1,201 (53.6)	
**Physical function**			0.002			< 0.001
Normal	226 (39.9)	137 (30.5)		420 (40.3)	233 (19.4)	
Dysfunction	341 (60.1)	312 (69.5)		621 (59.7)	968 (80.6)	
**ADL**			< 0.001			< 0.001
Normal	364 (64.2)	219 (48.8)		527 (50.6)	497 (41.4)	
Disability	203 (35.8)	230 (51.2)		514 (49.4)	704 (58.6)	

In 2015, the older adults with depressive symptoms had higher ADL disability (51.5%) than those without depressive symptoms (40.9%) in urban areas (Table 2). In 2018, the proportion of ADL disability with depressive symptoms (51.2) was higher than for older adults without depressive symptoms (35.8%) (Table 3). The percentage of older adults with physical dysfunction who were depressed was 69.5% in 2018 compared to 81.5% in 2015.

In rural areas, older adults who had trouble taking care of themselves were more likely to be depressed. In 2015, 90.3% of rural older adults with physical dysfunction had elevated depressive symptoms, and in 2018, 80.6% had elevated depressive symptoms. The percentage of depressed older adults with ADL disability was 68.9% in 2015 compared to 58.6% in 2018. In both 2015 and 2018, older adults with ADL disability had higher rates of depressive symptoms than those without depressive symptoms (Tables 2, 3).

### 3.4. Association between physical function, ADL, and depressive symptoms

Table 4 depicts the relationship between physical function and ADL and depressive symptoms in urban and rural older adults in 2015. Table 5 describes the relationship between physical function and ADL and depressive symptoms in 2018 urban and rural populations of older adults. In 2015 and 2018, we found that ADL disability was significantly associated with depressive symptoms among older adults in both urban and rural areas.

**Table 4:** OR with 95% CI of depressive symptoms according to the physical function and ADL stratified by urban and rural in 2015.

**Variables**		**Urban**		**Rural**	
		**Crude OR (95% CI)**	**Adjusted OR (95% CI)**	**Crude OR (95% CI)**	**Adjusted OR (95% CI)** ^a^
Physical function	Normal	Ref		Ref	
	Dysfunction	0.93 (0.64–1.35)	0.95 (0.65–1.39)	1.48 (1.14–1.93)^**^	1.51 (1.15–1.98)^**^
ADL	Normal	Ref		Ref	
	Disability	1.55 (1.16–2.07)^**^	1.50 (1.11–2.03)^**^	1.89 (1.59–2.26)^***^	1.69 (1.40–2.03)^***^

^a^Adjusted for gender, age, marital status, smoking, drinking, and self-rated health.

^*^P < 0.05, ^**^P < 0.01, and ^***^P < 0.001.

**Table 5:** OR with 95% CI of depressive symptoms according to the physical function and ADL stratified by urban and rural in 2018.

**Variables**		**Urban**		**Rural**	
		**Crude OR (95% CI)**	**Adjusted OR (95% CI)**	**Crude OR (95% CI)**	**Adjusted OR (95% CI)** ^a^
Physical function	Normal	Ref		Ref	
	Dysfunction	1.52 (1.17–1.98)	1.22 (0.78–1.89)	2.75 (2.28–3.33)^***^	1.61 (1.20–2.17)^**^
ADL	Normal	Ref		Ref	
	Disability	1.89 (1.47–2.44)^***^	1.79 (1.38–2.32)^***^	1.38(1.16–1.98)^***^	1.34 (1.13–1.60)^**^

^a^Adjusted for gender, education level, marital status, drinking, physical exercise, and self-rated health.

^*^P < 0.05, ^**^P < 0.01, and ^***^P < 0.001.

In urban areas, ADL disability was associated with a higher risk of depressive symptoms in 2015 (OR = 1.50) and in 2018 (OR = 1.79). In rural areas, ADL disability (OR = 1.69) and physical dysfunction (OR = 1.51) were associated with a higher risk of depressive symptoms in 2015. Similarly, in 2018, ADL disability (OR = 1.34) and physical dysfunction (OR = 1.61) were significant (Tables 4, 5).

In summary, both ADL disability and physical dysfunction were more likely to be associated with depressive symptoms in rural older adults.

## 4. Discussion

Based on data from the China Longitudinal Survey of Health and Retirement (CHARLS) in 2015 and 2018, we compared the characteristic differences among populations of depressive symptoms in older adults. In addition, multivariate logistic regression models were designed to identify urban-rural differences in physical function, ADL, and depressive symptoms in the Chinese adults, and to adjust for confounding factors. Key findings of the present study were (1) the prevalence of depressive symptoms among older adults in China was higher in 2015 than in 2018, and (2) residence, gender, marital status, drinking, physical function, ADL, and self-rated health were linked to depressive symptoms, and (3) among rural older adults with ADL disability and physical dysfunction, the likelihood of depressive symptoms was higher.

In the current report, the prevalence of depressive symptoms among older adults in China varied from 33.8% in 2015 to 50.6% in 2018, indicating a high level of depressive symptoms. The results were like previous research, depressive symptoms burden had been and would be progressively enhancing in China (7, 27). One study demonstrated that depressive symptoms were over 41% among older adults in China (28). A study in Bangladesh surveyed 168 healthy retired residents aged 60-80 years and found a 36.9% rate of depressive symptoms in older adults (29). While in a cross-sectional study abroad, the rate of depressive symptoms was 66.9% in 229 older adults in Hanoi, Vietnam (30).

Depressive symptoms were higher among rural older adults in our survey than among urban older adults. With rapid social and economic development, the gap between urban and rural areas has become more pronounced. Young and middle-aged workers work in municipalities, while older people and children live in rural areas. More attention should be paid to the mental health of older adults (31). A previous study identified that older adults who live alone in rural areas have a higher risk of depressive symptoms (31). “Empty nesters” tendency might be to account for the increased prevalence of depressive symptoms among older adults in rural China (32). The value of family was very important to the Chinese adults. Children of older adults in rural areas went out to work and were separated from their parents, reducing contact with the elderly, and increasing loneliness (33). At the same time, the responsibility for caring for infants among older adults in rural areas has expanded. As a result, older adults in rural China need additional social assistance. The health of older adults was considerably affected by the social environment. Urban older adults had higher quality medical resources and financial assistance than rural older adults (34). Older adults in urban areas can enjoy social activities and find spiritual comfort in their spare time. These findings denote that the government and society should pay more attention to the psychological problems of older adults in rural areas, allocate resources more effectively, expand public service provision, and reduce the gap between urban and rural areas (35).

In addition, the results demonstrated that residence, gender, marital status, drinking, physical function, ADL, and self-rated health were linked to depressive symptoms. Females were more likely to experience depressive symptoms. According to a Chinese study on the relationship between fat and depressive symptoms, 19.9% of males and 33.2% of females had depressive symptoms. Females were more likely to be depressed as a result of hormonal variations (36). According to a longitudinal study of aging in Ireland, females had a greater fear of tumbling and activity restrictions. This fear could affect the psychology of older adults. Our findings agreed with previous studies. In our study, older adults who were married had lower rates of depressive symptoms. Previous research on older adults has revealed that marital status was a strong predictor of depressive symptoms, with unmarried older adults being more likely to be depressed (6, 37). Single or split older adults had higher levels of depressive symptoms (37). Older adults could be psychologically affected by these events. Alcohol use and self-rated health were shown to be strongly linked with depressive symptoms in older adults in a poll of community-dwelling older adults (38). According to the survey results, self-rated health was highly correlated with depressive symptoms in older adults, which was consistent with earlier studies. A prospective study conducted in Spanish uncovered that moderate alcohol use protects older adults from developing depressive symptoms (39). Furthermore, Dao A et al. indicated that elderly people who drank alcohol had 3.4 times fewer depressive symptoms than no-drinkers (30). The second most important factor in determining depressive symptoms was self-rated health. Evidence was mounting that the older adults who self-rate their health as poor had higher levels of depressive symptoms (6). In the 2015 findings, older adults aged 60-70 and non-smokers were more likely to be depressed. While not significant in the 2018 study results. The two-year sample size varied, as did the study's findings. Further investigation was needed in the future to reveal the relationship between smoking and age and depressive symptoms.

Ultimately, this study focused on the relationship between physical function, ADL, and depressive symptoms in older Chinese adults in urban and rural areas. Physical function declines with age and numerous daily activities are difficult to perform independently. Physical decline was a key challenge to self-care ability of older adults (40). Limitations in daily activities and physical function cause older adults to lose their independence, leading to depressive symptoms and grief. These conditions could lead to psychosocial and financial difficulties. Substantial evidence suggested that ADL disability were at a higher risk of depressive symptoms (6), physical dysfunction associated with depressive symptoms in Chinese adults aged 55 and older (41). Older adults with elevated levels of functional restriction might have depressive symptoms (40), ADL disability might promote the development of depressive symptoms (22). This study demonstrates previous research by analyzing the association between physical function, ADL, and depressive symptoms. Depressed older adults were more likely to have physical dysfunction and ADL disability. Other studies have identified a strong association between ADL disability and physical dysfunction and risk of depressive symptoms in rural older adults. Therefore, the Chinese government and society must pay attention to the physical health of the elderly, especially those in rural areas. The government and society should give additional help to older adults with ADL disability and physical dysfunction. For individuals, sedentary lifestyle led to a decline in the capacity to conduct ADL (42). Older adults would require frequent physical activity to enhance their functional capacity and mental health (43).

Several limitations of the present study should be mentioned. First, the cross-sectional study was unable to draw causal inferences. Second, the CES-D-10 might exhibit recall bias and could only be used to screen for depressive symptoms, not to diagnose depressive symptoms (22). Third, the older adults included in this paper were screened from a database containing 23,000 respondents and may differ from the original data. Finally, self-aggregated data might overestimate the association between variables and depressive symptoms.

## 5. Conclusion

In summary, this study provides evidence of an association between physical function, ADL, and depressive symptoms in older Chinese adults. It showed that rural, female, 60–70 years old, primary school or below, married, non-smoking, non-drinking, physical dysfunction, ADL disability and self-rated poor health make-up a higher proportion of depressed older adults. Multivariate logistic regression models suggest that ADL disability and physical dysfunction were more likely to be associated with depressive symptoms in rural Chinese older adults. Older adults should be encouraged to participate in moderate physical and social activities to prevent physical dysfunction. The government and society should pay attention to the mental health of older adults in rural areas.

## Data availability statement

The original contributions presented in the study are included in the article/supplementary material, further inquiries can be directed to the corresponding author.

## Author contributions

YY and YD: design of the study and interpretation of data. XL: data processing. WP: data processing, article design, and revision. YC: article modification. All authors contributed to the article and approved the submitted version.

## References

[1] Li N Pang L Chen G Song X Zhang J Zheng X. Risk factors for depression in older adults in Beijing. Can J Psychiatry. (2011) 56:466–73. 10.1177/070674371105600804 21878157

[2] Schaakxs R Comijs HC Lamers F Kok RM Beekman ATF Penninx BWJH. Associations between age and the course of major depressive disorder: a 2-year longitudinal cohort study. Lancet Psychiatry. (2018) 5:581–90. 10.1016/S2215-0366(18)30166-4 29887519

[3] Kaneko Y Motohashi Y Sasaki H Yamaji M. Prevalence of depressive symptoms and related risk factors for depressive symptoms among elderly persons living in a rural Japanese community: a cross-sectional study. Community Ment Health J. (2007) 43:583–90. 10.1007/s10597-007-9096-5 17619147

[4] Tengku Mohd TAM Yunus RM Hairi F Hairi NN Choo WY. Social support and depression among community dwelling older adults in Asia: a systematic review. BMJ Open. (2019) 9:e026667. 10.1136/bmjopen-2018-026667 31320348 PMC6661578

[5] Campos AC Albala C Lera L Sánchez H Vargas AM. Ferreira e Ferreira E. Gender differences in predictors of self-rated health among older adults in Brazil and Chile. BMC Public Health. (2015) 15:365. 10.1186/s12889-015-1666-9 25884800 PMC4432978

[6] Fan X Guo X Ren Z Li X He M Shi H. The prevalence of depressive symptoms and associated factors in middle-aged and elderly Chinese people. J Affect Disord. (2021) 293:222–8. 10.1016/j.jad.2021.06.044 34217959

[7] Charlson FJ Baxter AJ Cheng HG Shidhaye R Whiteford HA. The burden of mental, neurological, and substance use disorders in China and India: a systematic analysis of community representative epidemiological studies. Lancet. (2016) 388:376–89. 10.1016/S0140-6736(16)30590-6 27209143

[8] Djernes JK. Prevalence and predictors of depression in populations of elderly: a review. Acta Psychiatr Scand. (2006) 113:372–87. 10.1111/j.1600-0447.2006.00770.x 16603029

[9] Kvæl LAH Bergland A Telenius EW. Associations between physical function and depression in nursing home residents with mild and moderate dementia: a cross-sectional study. BMJ Open. (2017) 7:e016875. 10.1136/bmjopen-2017-016875 28729326 PMC5541615

[10] Turvey CL Schultz SK Beglinger L Klein DM A. longitudinal community-based study of chronic illness, cognitive and physical function, and depression. Am J Geriatr Psychiatry. (2009) 17:632–41. 10.1097/JGP.0b013e31819c498c 19634203 PMC3690465

[11] Friedman MM Griffin JA. Relationship of physical symptoms and physical functioning to depression in patients with heart failure. Heart Lung. (2001) 30:98–104. 10.1067/mhl.2001.114180 11248712

[12] Stegenga BT Nazareth I Torres-González F Xavier M Svab I Geerlings MI. Depression, anxiety and physical function: exploring the strength of causality. J Epidemiol Community Health. (2012) 66:e25. 10.1136/jech.2010.128371 21693471

[13] Zhao D Hu C Chen J Dong B Ren Q Yu D. Risk factors of geriatric depression in rural China based on a generalized estimating equation. Int Psychogeriatr. (2018) 30:1489–97. 10.1017/S1041610218000030 29380720

[14] Bowen ME Ruch A. Depressive symptoms and disability risk among older white and latino adults by nativity status. J Aging Health. (2015) 27:1286–305. 10.1177/0898264315580121 25953809

[15] Yaka E Keskinoglu P Ucku R Yener GG Tunca Z. Prevalence and risk factors of depression among community dwelling elderly. Arch Gerontol Geriatr. (2014) 59:150–4. 10.1016/j.archger.2014.03.014 24767692

[16] Bozo O Toksabay NE Kürüm O. Activities of daily living, depression, and social support among elderly Turkish people. J Psychol. (2009) 143:193–205. 10.3200/JRLP.143.2.193-206 19306681

[17] Byeon H. Development of a depression in Parkinson's disease prediction model using machine learning. World J Psychiatry. (2020) 10:234–44. 10.5498/wjp.v10.i10.234 33134114 PMC7582129

[18] Bock JO Brettschneider C Weyerer S Werle J Wagner M Maier W. Excess health care costs of late-life depression–results of the AgeMooDe study. J Affect Disord. (2016) 199:139–47. 10.1016/j.jad.2016.04.008 27104802

[19] Kong D Solomon P Dong X. Depressive symptoms and onset of functional disability over 2 years: a prospective cohort study. J Am Geriatr Soc. (2019) 67:S538–S544. 10.1111/jgs.15801 31403199 PMC9942515

[20] Ni Y Tein JY Zhang M Yang Y Wu G. Changes in depression among older adults in China: A latent transition analysis. J Affect Disord. (2017) 209:3–9. 10.1016/j.jad.2016.11.004 27866046

[21] Jiang J Tang Z Futatsuka M. The impact of ADL disability on depression symptoms in a community of Beijing elderly, China. Environ Health Prev Med. (2002) 7:199–204. 10.1007/BF02898005 21432278 PMC2723587

[22] He M Ma J Ren Z Zhou G Gong P Liu M. et al. Association between activities of daily living disability and depression symptoms of middle-aged and older Chinese adults and their spouses: a community based study. J Affect Disord. (2019) 242:135–42. 10.1016/j.jad.2018.08.060 30173061

[23] Wang C Yang LS Shi XH Yang YF Liu K Liu RY. Depressive symptoms in aged Chinese patients with silicosis. Aging Ment Health. (2008) 12:343–8. 10.1080/13607860802120938 18728947

[24] Mengqi L. Influencing Factors and Policy Suggestions of Mental Health of Chinese Elderly–Based on the 2015 China Health and Retirement Longitudinal Study. Hangzhou, China: Zhejiang University. (2018)

[25] Chen H Mui AC. Factorial validity of the center for epidemiologic studies depression scale short form in older population in China. Int Psychogeriatr. (2014) 26:49–57. 10.1017/S1041610213001701 24125553

[26] Boey KW. Cross-validation of a short form of the CES-D in Chinese elderly. Int J Geriatr Psychiatry. (1999) 14:608–17. 10489651 10.1002/(sici)1099-1166(199908)14:8<608::aid-gps991>3.0.co;2-z

[27] Yang G Wang Y Zeng Y Gao GF Liang X Zhou M. Rapid health transition in China, 1990-2010: findings from the Global Burden of Disease Study 2010. Lancet. (2013) 381:1987–2015. 10.1016/S0140-6736(13)61097-1 23746901 PMC7159289

[28] Xie T Liu D Guo J Zhang B. The longitudinal effect of sensory loss on depression among Chinese older adults. J Affect Disord. (2021) 283:216–22. 10.1016/j.jad.2021.01.081 33561802

[29] Disu TR Anne NJ. Griffiths MD, Mamun MA. Risk factors of geriatric depression among elderly Bangladeshi people: a pilot interview study. Asian J Psychiatr. (2019) 44:163–9. 10.1016/j.ajp.2019.07.050 31382211

[30] Dao ATM Nguyen VT Nguyen HV Nguyen LTK. Factors associated with depression among the elderly living in urban Vietnam. Biomed Res Int. (2018) 2018:2370284. 10.1155/2018/2370284 30596085 PMC6286754

[31] Gong F Zhao D Zhao Y Lu S Qian Z Sun Y. The factors associated with geriatric depression in rural China: stratified by household structure. Psychol Health Med. (2018) 23:593–603. 10.1080/13548506.2017.1400671 29126351

[32] Purtle J Nelson KL Yang Y Langellier B Stankov I Diez Roux AV. Urban-rural differences in older adult depression: a systematic review and meta-analysis of comparative studies. Am J Prev Med. (2019) 56:603–13. 10.1016/j.amepre.2018.11.008 30777704

[33] Huang LJ MM RN Du WT MM, RN Liu YC MM, RN. Loneliness, stress, and depressive symptoms among the chinese rural empty nest elderly: a moderated mediation analysis. Issues Ment Health Nurs. (2019) 40:73–8. 10.1080/01612840.2018.1437856 30633625

[34] Selvaratnam DP Tin PB. Lifestyle of the elderly in rural and urban Malaysia. Ann N Y Acad Sci. (2007) 1114:317–25. 10.1196/annals.1396.025 17986592

[35] Liu D Xi J Hall BJ Fu M Zhang B Guo J. Attitudes toward aging, social support and depression among older adults: difference by urban and rural areas in China. J Affect Disord. (2020) 274:85–92. 10.1016/j.jad.2020.05.052 32469837

[36] Qian J Li N Ren X. Obesity and depressive symptoms among Chinese people aged 45 and over. Sci Rep. (2017) 7:45637. 10.1038/srep45637 28378748 PMC5381219

[37] Fatima M Sehar A Ali M Iqbal A Shaukat F. Incidence of depression among community dwelling healthy elderly and the predisposing socio-environmental factors. Cureus. (2019) 11:e4292. 10.7759/cureus.4292 31183273 PMC6538234

[38] Han KM Han C Shin C Jee HJ An H Yoon HK. Social capital, socioeconomic status, and depression in community-living elderly. J Psychiatr Res. (2018) 98:133–40. 10.1016/j.jpsychires.2018.01.002 29351862

[39] García-Esquinas E Ortolá R Galán I Soler-Vila H Laclaustra M Rodríguez-Artalejo F. Moderate alcohol drinking is not associated with risk of depression in older adults. Sci Rep. (2018) 8:11512. 10.1038/s41598-018-29985-4 30065286 PMC6068095

[40] Muramatsu N Yin H Hedeker D. Functional declines, social support, and mental health in the elderly: does living in a state supportive of home and community-based services make a difference? Soc Sci Med. (2010) 70:1050–8. 10.1016/j.socscimed.2009.12.005 20117865 PMC3360961

[41] Deng Y Paul DR. The relationships between depressive symptoms, functional health status, physical activity, and the availability of recreational facilities: a rural-urban comparison in middle-aged and older Chinese adults. Int J Behav Med. (2018) 25:322–30. 10.1007/s12529-018-9714-3 29498014

[42] Penha JC Piçarro Ida C de Barros Neto TL. Evolução da aptidão física e capacidade funcional de mulheres ativas acima de 50 anos de idade de acordo com a idade cronológica, na cidade de Santos. Cien Saude Colet. (2012) 17:245–53. 10.1590/S1413-81232012000100027 22218558

[43] Zaleski AL Taylor BA Panza GA Wu Y Pescatello LS Thompson PD. Coming of age: considerations in the prescription of exercise for older adults. Methodist Debakey Cardiovasc J. (2016) 12:98–104. 10.14797/mdcj-12-2-98 27486492 PMC4969034

