# Erratum: Physical function, ADL, and depressive symptoms in Chinese elderly: evidence from the CHARLS

**DOI:** 10.3389/fpubh.2023.1241226

**Published:** 2023-07-11

**Authors:** Frontiers Production Office

**Affiliations:** Frontiers Media SA, Lausanne, Switzerland

**Keywords:** ADL disability, physical dysfunction, depressive symptoms, urban and rural elderly, CHARLS

Due to a production error, an additional affiliation was incorrectly attributed to corresponding author Weiwei Ping.

Their correct affiliation is:

^2^Department of Public Health and Preventive Medicine, Changzhi Medical College, Changzhi, China

The publisher apologizes for this mistake. The original article has been updated.

